# Effect of various types of milk on salivary pH among children: a pilot randomized controlled crossover trial

**DOI:** 10.1038/s41405-023-00170-8

**Published:** 2023-09-13

**Authors:** Rouaa Zamzam, Mawia Karkoutly, Nada Bshara

**Affiliations:** Pediatric Dentistry Department, Faculty of Dentistry, Damascus University, Damascus, Syria

**Keywords:** Nutrition and diet in dentistry, Oral hygiene

## Abstract

**Objective:**

This study aimed to evaluate salivary pH changes after consuming three types of milk in children aged 3–5 years. The null hypothesis was that no statistically significant difference would be noted in the salivary pH between high-protein, full-fat, and sweetened milk groups at different time points.

**Materials and methods:**

This was a double-blind, pilot randomized controlled crossover trial. 30 Children have undergone three experimental sessions with a 1-week washout period. Each child was given 250 mL of one of the following types of milk: high-protein, full-fat, or sweetened milk. The salivary pH was measured at the baseline (t_0_) after 5 (t_1_), 10 (t_2_), 15 (t_3_), 30 (t_4_), and 60 (t_5_) minutes of milk consumption, using a pH saliva indicator strip.

**Results:**

There is a sharp drop in salivary pH after 5 min of sweetened (*P* < 0.05) and full-fat milk consumption (*p* < 0.05). However, the initial drop in the salivary pH was found to remain above the critical level. For the high-protein milk group, salivary pH decreased slightly after 5 min but was similar to that at the baseline (*p* = 0.573). In the high-protein milk group (*p* < 0.05), the salivary pH was slightly greater than the baseline value at t_5_.

**Conclusions:**

The study shows an initial suggestion that milk is a non-cariogenic beverage, even when sugar is added. Furthermore, high-protein milk has a protective effect from dental caries.

## Introduction

Milk is a liquid produced by mammals to meet the nutritional needs of newborns, and it is considered the primary source of nourishment in proper proportions [1]. In addition, it is considered non-cariogenic due to the high levels of calcium and phosphorus that exhibit antimicrobial properties and increase the salivary pH [1, 2]. Although milk contains lactose, which is moderately cariogenic, lactose is the least cariogenic fermentable sugar [2]. About 18.1% of daily protein intake is provided by milk and dairy products [3]. High-protein milk contains 100% skimmed cow milk and 6 g of protein per 100 mL [4, 5]. Protein is the most abundant organic compound throughout the body, and it is responsible for the buffering properties of saliva [6]. Whole milk contains 3.5% of fat [7], and children that are younger than 2 years should be provided with full-fat milk and then move to reduced-fat milk [8]. For infants, breast milk and formula milk are the primary sources of dietary fat, while older children get fat from animal products and vegetable oils [9]. Whole milk contains 0.04 g of omega-3 polyunsaturated fatty acids (n-3 PUFAs) per 237 mL or in one cup [10]. Fat provides essential fatty acids, which have a functional and structural role in the body [11]. Flavored milk is a dairy drink that is usually sweetened and contains 9.1 g of sugar per 100 mL [12]. Flavored milk is widely acceptable among children since they consume flavored milk more than plain milk [13]. However, flavored milk is highly processed and contains additives with no nutritional value. In addition, it exhibits a cariogenic potential due to its high sugar content [14].

Human saliva consists of more than 99% water and less than 1% organic and inorganic substances. The flow rate of unstimulated saliva is about 0.3–0.4 ml/min [15], and the normal pH range of saliva is 6.2–7.6 [16]. The salivary flow and saliva pH increase during chewing [17]. However, the salivary pH decreases after consuming cariogenic food, reaching the critical pH (pH = 5.5) or less, at which enamel demineralization begins [18]. Salivary buffer capacity aims to protect teeth from dental caries through the neutralization of plaque acids and products of acidogenic microorganisms. The normal range of salivary buffer capacity is estimated to be 3–30 mg/100 mL [19]. Milk has remineralization potential due to the high content of calcium, phosphorous, and casein phosphopeptides [2, 3]. Therefore, this study aimed to evaluate salivary pH changes after consuming high-protein, full-fat, and sweetened milk in children aged 3–5 years. The null hypothesis was that no statistically significant difference would be noted in the salivary pH between high-protein, full-fat, and sweetened milk groups at different time points.

## Materials and methods

### Study design and ethics

This was a double-blind, pilot randomized controlled crossover trial where each participant would be their comparator. It was conducted for 2 months at Damascus University. Ethical approval was provided by the Institutional Review Board at Damascus University (N7748), and it was conducted in full accordance with the 1964 Helsinki Declaration [20] and CONSORT statement [21]. Written informed consent was obtained from the participants’ legal guardians. The current trial was registered at Australian New Zealand Clinical Trials Registry (ACTRN12623000773639).

### Sample size calculation

Sample size was calculated using G*Power software 3.1.9.4 (Heinrich- Hein-Universitat-Dusseldorf, Germany; http://www.gpower. hhu.de/). Effect size f = 0.5996822/α err prob = 0.05/ Power (1-β err prob) = 0.80/ Number of groups = 3/ Total sample size = 30. Effect size was estimated according to the results of a pilot study of five participants, and a sample size of 30 children was sufficient to obtain an effect size of 0.59.

### Recruitment and eligibility criteria

#### Inclusion criteria

1. Healthy children that are free of systemic diseases.
2. Children with good oral hygiene, according to Oral Hygiene Index (OHI) [22].
3. Children aged 3–5 years.
4. Children who are definitely positive, or positive according to Frankl’s behavior rating scale.
5. Children with full primary dentition.

#### Exclusion criteria

1. Taking medications that affect the salivary flow rate.
2. Intolerance to milk protein.
3. Children undergoing orthodontic treatment.

**Fig. 1:**
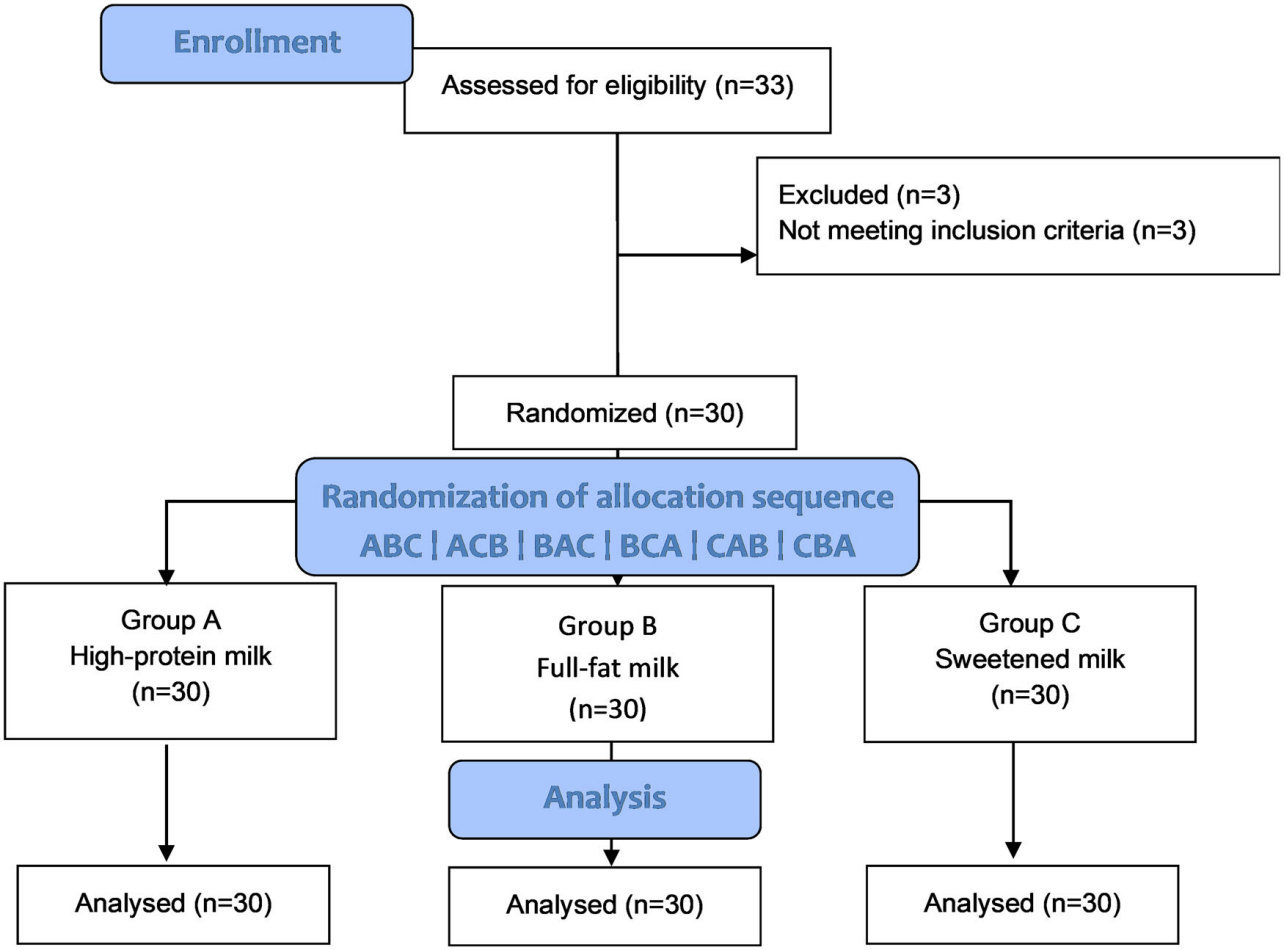
Modified CONSORT flowchart.

33 patients were assessed for eligibility, and 3 were excluded due to not meeting the inclusion criteria. The modified CONSORT flowchart is presented in Fig. 1.

### Intervention

Children have undergone three experimental sessions with a 1-week washout period [23]. The sequence of the milk types for the three interval sessions was randomized using a computer algorithm. Children were instructed not to brush their teeth, eat, and drink for 2 h before this study [24]. Salivary pH was recorded at the baseline (t_0_) then each child was given 250 mL of one of the following types of milk:

1. High-protein milk (High Protein Milk, Hawa Al Sham Dairies & Cheese Co., Damascus, Syria).
2. Full-fat milk (Long Life Milk, Hawa Al Sham Dairies & Cheese Co., Damascus, Syria).
3. Sweetened milk (Chocolate Flavored Milk, Hawa Al Sham Dairies & Cheese Co., Damascus, Syria).

The salivary pH was measured after 5 (t_1_), 10 (t_2_), 15 (t_3_), 30 (t_4_), and 60 (t_5_) minutes of milk consumption. Salivary pH was measured using a pH saliva indicator strip (Duotest pH 3.5–6.8, Macherey-Nagel® GmbH & Co. KG, Düren, Germany), which is to Alkarad et al. [25] study. Children were instructed to allow saliva to flow passively in the mouth for 5 min at the six-time points then they were asked to split 2 mL of saliva into a graduated tube. The pH saliva indicator strip was immersed in saliva for 1 min then the color change was recorded and evaluated according to the reference color chart [25]. Both the data assessor and the statistician were blinded to group allocations.

The composition of the milk samples according to the manufacturing industry were as follows:

1. High-protein milk (protein: 25 g/100 mL, fat: 3 g/100 mL, and carbohydrate: 5.1 g/100 mL).
2. Full-fat milk (protein: 3.1 g/100 mL, fat: 3 g/100 mL, and carbohydrate: 5.1 g/100 mL).
3. Sweetened milk (protein: 3.1 g/100 mL, fat: 0.5 g/100 mL, and carbohydrate: 11 g/100 mL).

The starting pH of the milk samples was tested using pH meter (WTW pH/Ion 340i meter, Xylem Analytics Germany Sales GmbH & Co. KG, WTW., Weilheim, Germany). The starting pH was as follows:

1. High-protein milk (pH = 5.84).
2. Full-fat milk (pH = 5.82).
3. Sweetened milk (pH = 5.51).

### Statistical analysis

Statistical analysis was performed using IBM SPSS software version 24 (IBM Corp., Armonk, USA). Descriptive statistics were calculated and provided as mean, standard deviation, standard error, minimum, and maximum. Kurskal–Wallis test was performed to compare non-paired data, and Wilcoxon signed-rank test was used to compare paired data. Statistical significance was adjusted at 0.05 (*p* < 0.05).

## Results

A total of 33 patients were assessed for eligibility, and 3 were excluded due to not meeting the inclusion criteria. The sample obtained was 30 children, and more than half of them, were male (*n* = 20; 67%). The mean age was 4 years (SD 0.81; range 3–5 years). The mean of the dmft score was 4.8 ± 3.9. The salivary pH values were listed as mean, standard deviation (SD), standard error (SE), minimum (Min), and maximum (Max) for each group at different time points (Table 1).

**Table 1:** Descriptive statistics of salivary pH at different time points of study groups.

Group	*n*	Time point	Mean ± SD	SE	Min	Max
High-protein milk	30	t_0_	6.07 ± 0.33	0.06	5.3	6.8
		t_1_	6.05 ± 0.35	0.06	5.6	7
		t_2_	6.06 ± 0.27	0.05	5.6	6.8
		t_3_	6.12 ± 0.22	0.04	5.9	6.5
		t_4_	6.16 ± 0.26	0.05	5.6	6.5
		t_5_	6.18 ± 0.29	0.05	5.6	6.8
Full-fat milk	30	t_0_	6.12 ± 0.30	0.06	5.6	6.5
		t_1_	5.81 ± 0.24	0.04	5.6	6.2
		t_2_	5.97 ± 0.20	0.04	5.6	6.5
		t_3_	6.02 ± 0.23	0.04	5.6	6.5
		t_4_	6.12 ± 0.25	0.05	5.6	6.5
		t_5_	6.18 ± 0.26	0.05	5.6	6.5
Sweetened milk	30	t_0_	6.20 ± 0.32	0.06	5.6	6.8
		t_1_	5.56 ± 0.17	0.03	5.3	5.9
		t_2_	5.72 ± 0.15	0.03	5.6	5.9
		t_3_	5.86 ± 0.15	0.03	5.6	6.2
		t_4_	6.01 ± 0.20	0.04	5.6	6.2
		t_5_	6.10 ± 0.21	0.04	5.6	6.5

No statistically significant difference was found between the mean salivary pH values at t0 (*p* = 0.306), suggesting that the baseline data are well-balanced. However, the Kruskal–Wallis test indicated a significant difference between the study groups at t_1_, t_2_, t_3_, and t_4_ (*p* < 0.05) (Table 2). The pairwise comparison test indicated a significant difference between high-protein milk and full-fat milk groups at t_1_ (*p* < 0.05) and a significant difference between high-protein milk and sweetened milk groups at t_1_, t_2_, t_3_, and t_4_ (*p* < 0.05). In addition, there was a statistically significant difference between full-fat milk and sweetened milk groups at t_1_, t_2_, and t_3_ (*p* < 0.05) (Table 3).

**Table 2:** Results of Kruskal–Wallis test for comparison between the salivary pH at different time points in three groups.

Time points	Groups	Chi-square value	*p* value
t_0_	High-protein milk	2.367	0.306
	Full-fat milk		
	Sweetened milk		
t_1_	High-protein milk	35.616	<0.001*
	Full-fat milk		
	Sweetened milk		
t_2_	High-protein milk	33.054	<0.001*
	Full-fat milk		
	Sweetened milk		
t_3_	High-protein milk	21.284	<0.001*
	Full-fat milk		
	Sweetened milk		
t_4_	High-protein milk	6.275	0.043*
	Full-fat milk		
	Sweetened milk		
t_5_	High-protein milk	2.031	0.362
	Full-fat milk		
	Sweetened milk		

*Significant difference at *p* < 0.05.

**Table 3:** Pairwise comparison between groups at different time points.

Time points	Pairwise comparisons	Mean difference	*p* value
t_1_	High-protein milk vs. Full-fat milk	0.24	0.006*
	High-protein milk vs. Sweetened milk	−0.49	<0.001*
	Full-fat milk vs. Sweetened milk	−0.25	<0.001*
t_2_	High-protein milk vs. Full fat milk	0.09	0.148
	High-protein milk vs. Sweetened milk	−0.34	<0.001*
	Full-fat milk vs. Sweetened milk	−0.25	<0.001*
t_3_	High-protein milk vs. Full fat milk	0.10	0.059
	High-protein milk vs. Sweetened milk	−0.26	<0.001*
	Full-fat milk vs. Sweetened milk	−0.16	0.004*
t_4_	High-protein milk vs. Full fat milk	0.04	0.506
	High-protein milk vs. Sweetened milk	−0.15	0.016*
	Full-fat milk vs. Sweetened milk	−0.11	0.076*

*Significant difference at *p* < 0.05.

In the high-protein milk group, a significant difference was found in the salivary pH at t_4_ and t_5_ compared to t_0_ (*p* < 0.05). However, there was no significant difference in the salivary pH at t_1_ (*p* = 0.573), t_2_ (*p* = 0.876), and t_3_ (*p* = 0.222) compared to t_0_. In the full-fat milk group, a significant difference was noted in the salivary pH at t_1_, t_2_, and t_3_ (*p* < 0.05), but no significant difference was observed at t_4_ (*p* = 1.000) and t_5_ (*p* = 0.058) compared to t_0_. In the sweetened milk group, there was a significant difference at the five-time points compared to t_0_ (*p* < 0.05) (Table 4).

**Table 4:** Wilcoxon signed-rank test results for comparison of baseline salivary pH with different time points salivary pH within each group

Groups	Time points	Mean difference	*z* value	*p* value
High-protein milk	t_0_ vs. t_1_	−0.02	−0.564	0.573
	t_0_ vs. t_2_	−0.01	−0.156	0.876
	t_0_ vs. t_3_	0.05	−1.221	0.222
	t_0_ vs. t_4_	0.09	−2.310	0.021
	t_0_ vs. t_5_	0.11	−2.810	0.005*
Full-fat milk	t_0_ vs. t_1_	−0.31	−4.184	<0.001*
	t_0_ vs. t_2_	−0.15	−2.696	0.007*
	t_0_ vs. t_3_	−0.10	−2.352	0.019*
	t_0_ vs. t_4_	0.00	0	1.000
	t_0_ vs. t_5_	0.06	−1.897	0.058
Sweetened milk	t_0_ vs. t_1_	−0.64	−4.915	<0.001*
	t_0_ vs. t_2_	−0.48	−4.657	<0.001*
	t_0_ vs. t_3_	−0.34	−4.582	<0.001*
	t_0_ vs. t_4_	−0.19	−3.788	<0.001*
	t_0_ vs. t_5_	−0.10	−2.486	0.013*

*Significant difference at *p* < 0.05.

**Fig. 2:**
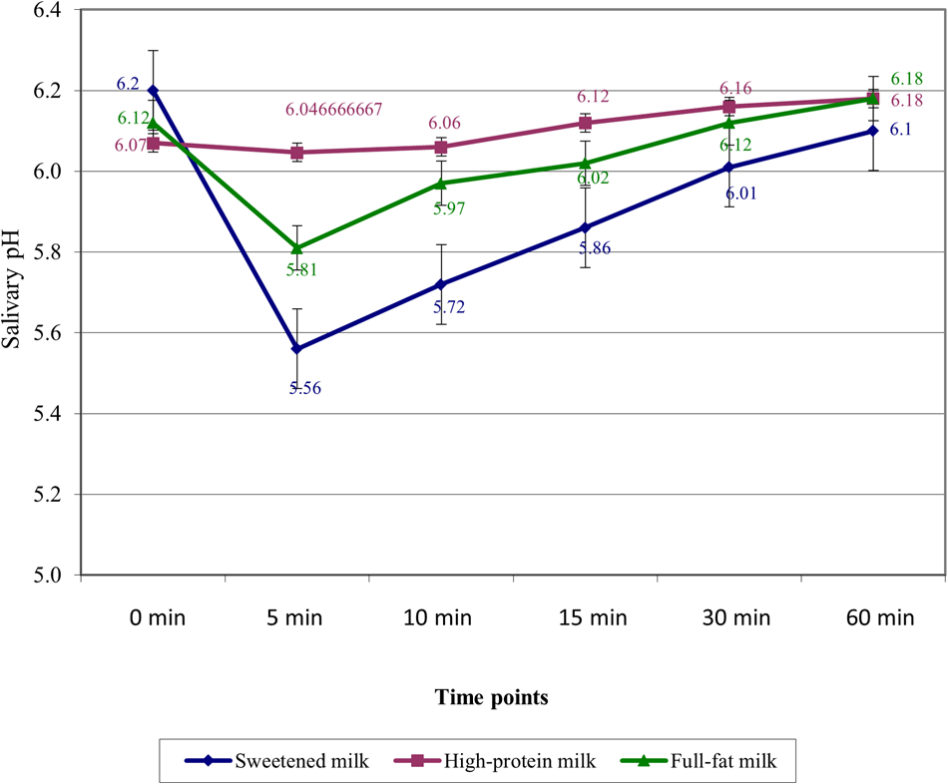
The Kinetics of salivary pH at different time points.

The Kinetics of salivary pH were presented in Fig. 2. There is a sharp drop in salivary pH after 5 min of sweetened and full-fat milk consumption. Subsequently, a gradual recovery was noted within 60 min. However, the initial drop in the salivary pH was found to remain above the critical level. For the high-protein milk group, salivary pH decreased slightly after 5 min but was similar to that at the baseline. In high-protein and full-fat milk groups, the salivary pH was slightly greater than the baseline value at t_5_.

## Discussion

Dental caries is the most common chronic disease in both children and adults. The frequent consumption of dietary free sugars between mealtimes increases the risk of dental caries, especially in schoolchildren [26]. To the best of the authors’ knowledge, no study has ever evaluated the cariogenic potential of various types of milk among children. Therefore, this study aimed to evaluate salivary pH changes after consuming high-protein, full-fat, and sweetened milk in children aged 3–5 years.

Salivary pH is a biomarker for many oral and systemic diseases, such as hypertension, insulin resistance, chronic kidney disease, depression, periodontal diseases, and dental caries. [27, 28] In addition, salivary pH is used to evaluate the cariogenic potential of certain foods and beverages [29]. In the current study, litmus paper was used for measuring salivary pH due to its high reliability and quick documentation [30], which is to Alkarad et al. [25] study. In this study, the milk intake was 250 mL, which is equivalent to one carton of milk [31]. In addition, the washout period was 1 week, which is to Barajas-Torres et al. [23] study.

In the high-protein milk group, salivary pH decreased slightly after 5 min but was similar to that at the baseline, and there was no significant difference in the salivary pH at t_1_, t_2_, and t_3_ compared to t_0_. In addition, the salivary pH was slightly greater than the baseline value at t_5_, and there was a significant difference in the salivary pH at t_4_ and t_5_ compared to t_0_. This result could be explained by the high protein content, which has a high buffering capacity. High protein concentration increases the salivary pH since it increases the flow rate of saliva [32, 33]. As a result, high-protein milk has a non-cariogenic potential and a protective effect from dental caries. This finding is in agreement with the one reported by Sulastri et al. [34], suggesting that high-protein milk has a high buffering capacity., and the salivary pH was measured using pH strip. In addition, according to Savira et al. [35], salivary pH value increase after consumption of Ultra high-temperature milk because of its high protein content.

For the full-fat milk group, there is an initial drop in salivary pH after 5 min of consumption, and a significant difference was noted in the salivary pH at t_1_, t_2_, and t_3_ compared to t_0_. Subsequently, a gradual recovery was noted within 60 min, and no significant difference was observed at t_4_ and t_5_ compared to t_0_. This could be explained by the fact that milk fat adsorbs to the surface of the enamel and may play a protective effect [32]. However, unlike this finding, Azrak et al. [36] suggested that, despite the caries-protective components, since of the lactose substance, milk can only be prescribed for constrained consumption. In addition, Junge et al. [37] found that plain milk can reduce Streptococcus mutans counts only when it is supplemented with probiotic.

The result of the current study suggested that there is a significant difference between high-protein milk and full-fat milk groups at t_1_. This finding is consistent with one reported by Seralurin et al. [38] suggesting that there is a decrease in the salivary pH value after consuming powdered cow milk, which has higher fat content compared to pure cow milk.

In the sweetened milk group, there is a sharp drop in salivary pH after 5 min of consumption. Subsequently, a gradual recovery was noted within 60 min and there was a significant difference at the five-time points compared to t_0_. This result is similarly reported by Hirani et al. [39]. However, according to Bhat et al. [40] study, the salivary pH reached the baseline level at 30 min after sweetened milk consumption. A possible explanation for this finding is that the mean dmft score was higher in the current study. However, the initial drop in the salivary pH was found above the critical level, which causes enamel demineralization [18]. As a result, the sweetened milk in this study is a non-cariogenic beverage. This finding is similar with Bhat et al. [40] study, suggesting that the salivary pH value returns to the baseline after 120 min of sweetened milk consumption. Navit et al. [41] found that adding flavors to milk does not cause a significant change in the salivary pH after their intake, but the significant drop was noted immediately after their consumption. Furthermore, Khodadadi et al. [42] reported that because of the antioxidant properties of certain additives, flavored milk could be recommended for children. Shah et al. [43] suggested that when sugar is added to milk, the salivary pH does not fall below the critical level. This result could be explained by the fact that milk has an anti-caries activity due to its content of casein phosphopeptides, phosphate, and calcium ions, which hinder demineralization. In addition, milk enzymes inhibit the proliferation of acidogenic bacteria [44]. Hence, milk has a protective effect from dental caries even when sugar is added. However, this finding is in contrast with the one reported by Bhure et al. [45] result suggesting that milk has a cariogenic potential when sugary additives are added to it. This could be attributed to the high carbohydrate content of Bourn Vita (17 g/100 mL), and Horlicks (14 g/100 mL) sugary additives, when compared with the carbohydrate content reported in the current study (11 g/100 mL).

This study has limitations. Cross-over trial designs are subjected to carryover effects, and it is hard to accurately estimate the washout period required. In addition, the effect of each type of milk was only tested once. As milk is not consumed only once by each child. Furthermore, litmus paper is not as accurate as pH meter [46].

## Conclusions

Within the limitations of this study, it shows an initial suggestion that milk is a non-cariogenic beverage, even when sugar is added. Furthermore, high-protein milk has a non-cariogenic potential and high buffering capacity. Hence, it has a protective effect from dental caries. Further trials with larger sample sizes are recommended to ascertain results. In addition, it would be appropriate to conduct an experiment where milk is consumed 3 times per day during a period of at least 7 days. Furthermore, it is recommended to use a microelectrode technology, which is more precise to evaluate the salivary pH changes. However, this method was not used due to its unavailability.

## Data Availability

The datasets used and/or analyzed during the current study are available from the corresponding author on reasonable request.
